# Development of optimization method for truss structure by quantum annealing

**DOI:** 10.1038/s41598-024-64588-2

**Published:** 2024-06-16

**Authors:** Rio Honda, Katsuhiro Endo, Taichi Kaji, Yudai Suzuki, Yoshiki Matsuda, Shu Tanaka, Mayu Muramatsu

**Affiliations:** 1 Graduate School of Science and Technology, Keio University, 3-14-1 Hiyoshi, Kohoku-ku, Yokohama, Kanagawa 223-8522 Japan; 2 Research Center for Computational Design of Advanced Functional Materials, National Institute of Advanced Industrial Science and Technology (AIST), 1-1-1 Umezono, Tsukuba, Ibaraki 305-8568 Japan; 3 Keio Quantum Computing Center, Keio University, 3-14-1 Hiyoshi, Kohoku-ku, Yokohama, Kanagawa 223-8522 Japan; 4 Fixstars, 3-1-1 Shibaura, Minato-ku, Tokyo 108-0023 Japan; 5 Green Computing System Research Organization, Waseda University, 27 Wasedacho, Shinjuku-ku, Tokyo 162-0042 Japan; 6 Department of Applied Physics and Physico-Informatics, Keio University, 3-14-1 Hiyoshi, Kohoku-ku, Yokohama, Kanagawa 223-8522 Japan; 7 Human Biology-Microbiome-Quantum Research Center (WPI-Bio2Q), Keio University, 35 Shinanomachi, Shinjuku-ku, Tokyo 160-8582 Japan; 8 Department of Mechanical Engineering, Keio University, 3-14-1 Hiyoshi, Kohoku-ku, Yokohama, Kanagawa 223-8522 Japan

**Keywords:** Civil engineering, Mechanical engineering, Computational science

## Abstract

In this study, we developed a new method of topology optimization for truss structures by quantum annealing. To perform quantum annealing analysis with real variables, representation of real numbers as a sum of random number combinations is employed. The nodal displacement is expressed with binary variables. The Hamiltonian *H* is formulated on the basis of the elastic strain energy and position energy of a truss structure. It is confirmed that truss deformation analysis is possible by quantum annealing. For the analysis of the optimization method for the truss structure, the cross-sectional area of the truss is expressed with binary variables. The iterative calculation for the changes in displacement and cross-sectional area leads to the optimal structure under the prescribed boundary conditions.

## Introduction

Topology optimization is the problem of finding a combination of components that provides high performance at a low cost, such as in designs of bridges, towers, and so on^1–3^. When trying to solve a combinatorial optimization problem such as topology optimization, it is possible to get stuck in a local solution during the computation. Therefore, repeated calculations are required with small steps to obtain a globally optimal solution^4^. Such small steps increase the computational cost, and thus computational methods that avoid getting stuck in local solutions are becoming increasingly important.

In recent years, quantum computers have been applied to solving some practical problems^5–10^. Quantum computers are attracting attention owing to their capability to perform calculations faster than classical computers for some problems.

Quantum computers use properties such as quantum superposition and quantum entanglement to achieve high-speed computation. A bit used in a classical computer represents a state of 0 or 1. In contrast, a qubit used in a quantum computer can handle a superposition of 0 and 1. This allows multiple states to be computed in parallel. Quantum entanglement also enables the handling of quantum interactions between distant qubits^11,12^. In a classical computer, the state of one bit does not affect the state of other bits, whereas in a quantum computer, a change in the state of one qubit can in conjunction change the state of other qubits. This interaction between different qubits is called quantum entanglement. This makes it possible to manipulate multiple interacting qubits simultaneously^13,14^.

**Figure 1:**
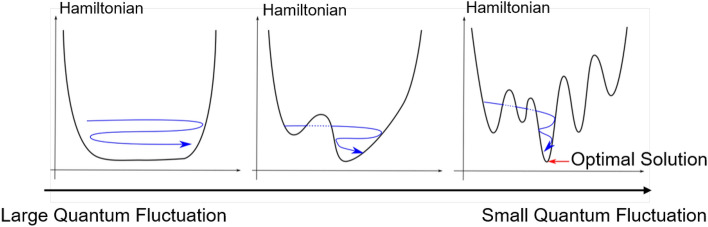
Schematic of the system of quantum annealing^14^.

There are two types of quantum computer, i.e., the quantum gate model^15–17^ and quantum annealing^18–20^. The quantum gate model has been studied more extensively than quantum annealing, and it is observed to be more efficient than classical computers for some calculations, such as simulations of quantum mechanics^21–27^. However, the large-scale circuits with a large number of qubits, which are the basic elements are still unstable. Quantum annealing, on the other hand, also uses qubits as basic elements and specializes in solving discrete optimization problems.

The difference between gate-based quantum computers and quantum annealers lies in the difference in the method of quantum computation. Gate-based quantum computers performs quantum computations by applying a series of quantum gates to manipulate the quantum state of qubits. Quantum annealers, on the other hand, are a physical implementation of the adiabatic theorem. The adiabatic theorem states that when a quantum state is the ground state in an initial Hamiltonian if the Hamiltonian changes slowly enough, the quantum state maintains the ground state of the Hamiltonian after the change. In quantum computers, the quantum states are disturbed by unwanted state changes caused by noise to the quantum system^28^. This is because it is difficult to isolate the qubits sufficiently from the effects of external noise, in contrast to a classical computer, which has a robust on/off state of transistor switches distinguished by billions of electrons^29^. For this reason, gate-based quantum computers generally cannot perform calculations robust to noise unless the error rate of the gate is kept below a certain level^30^. However, there are theoretically noise-tolerant computational schemes in quantum computers, and adiabatic quantum computation (AQC) is one of them. The reason why AQC is robust to noise is claimed in the literature^31^ as follows: (1) The phase of the ground state does not affect the effectiveness of the algorithm. (2) Transitions between eigenstates are problematic for AQC, but AQC is efficient only when the minimum energy gap of the Hamiltonian is not too small, and should be robust against decoherence in such situations. (3) If the error in the Hamiltonian due to noise changes slowly and the initial and final errors are not too large. The nature of AQC allows for reasonably large deviations from the original Hamiltonian. Since the quantum annealing machine is one implementation of AQC, the quantum annealing machines are expected to be robust against noise. In 2023, a quantum computer capable of handling more than 5,000 qubits was developed by D-Wave^21^.

Quadratic Unconstrained Binary Optimization (QUBO) is a type of combinatorial optimization problem that can be solved by quantum annealing. The following equation describes the Hamiltonian of QUBO:

1 $$\begin{aligned} H=-\sum _{i, j} Q_{i j} q_{i} q_{j}\quad \left( q_{i}=0,1\right) \end{aligned}$$

The Hamiltonian of QUBO is the sum of the product of two binary variables $q_{i}$ and $q_{j}$ multiplied by a constant $-Q_{i j}$. Converting the combinatorial optimization problem into QUBO Hamiltonian, it can be computed using quantum annealing^26,32–35^.

Quantum annealing is based on the principle of the tunneling effect caused by quantum fluctuations, which induces state transition, achieving the ground state. The ground state is finally achieved by the operation of quantum fluctuations, i.e., by first giving large quantum fluctuations and then gradually minimizing them. Figure 1 shows a schematic of quantum annealing. Quantum annealing has already been applied to solving discrete optimization problems such as traffic volume control and nurse scheduling^36–38^. Some studies have shown a marked increase in computational speed by a factor of approximately 100 million.

Recently, a method of solving linear systems called FEqa has been proposed for problems discretized by the finite element method^39^. In this method, the finite element problem is calculated using a classical computer, and quantum annealing is used to minimize the residuals. Quantum annealing may have more advantages in structural optimization problems. This is because quantum annealing finds the global optimal solution efficiently, which leads to a higher computation speed than in the case of using a classical computer.

The objective of this study is to develop an optimization method for truss structures by quantum annealing. To represent real numbers using binary variables, among various methods for representing the real numbers^40–45^, we employ the method of “combinatorial random number sums”^46^. The deformation analysis of a truss structure with quantum annealing is performed by expressing the nodal displacement with binary variables. The Hamiltonian is formulated on the basis of the elastic strain energy for infinitesimal deformation and the position energy of the truss structure. The optimization analysis of the truss structure is performed by quantum annealing by also expressing the cross-sectional area of the truss structure as a binary variable.

There are several prior studies that have used quantum annealing to optimize truss structures. In a previous study, a topology optimization method for quadrilateral elements using quantum annealing has been proposed^47,48^. On the other hand, our research aims to optimize the cross-sectional area of a truss, which is a different target problem. There is also research that transforms the problem of optimizing the cross-sectional area of a truss structure into a form that can be solved by quantum annealing using the principle of minimum potential energy in the form of order reduction^49^. This work requires the introduction of ancilla qubits to simultaneously optimize the displacement and the cross-sectional area. While this method has a formulation similar to the classical method, it cannot handle large problems. However, our method can handle large problems because it can be analyzed by annealing without using ancilla qubits by introducing an approximation for small deformations. In addition, this study employs binary sums for the representation of real numbers, but for complex problems, the correct solution may not be obtained. On the other hand, our study uses a random combinatorial sum representation of real numbers, which may lead to more accurate solutions^46^.

The objective of the optimization problem in this study is to find the truss structure with the lowest displacement and highest stiffness under the same boundary conditions, with the design condition being the cross-sectional area. To achieve this, the deformation analysis and the calculation of the cross-sectional area changes are performed using quantum annealing. In the deformation analysis, the objective function is first minimized and consists of the sum of the elastic strain energy of the truss and the energy due to external forces. A small deformation approximation is used to formulate the objective function using nodal displacements as variables. Then, the objective function, defined as the elastic strain energy of the truss, is maximized in the section on changes in the cross-sectional area. Additionally, a constraint is imposed to keep the total sum of the truss sectional areas constant, using sectional areas as the variables.

The variables used in the calculations in this study are displacements and cross-sectional areas, which are continuous variables, while the variables that can be used in quantum annealing are binary variables. Therefore, it is necessary to represent real numbers using binary variables. In this study, real numbers are represented using the method called the combinatorial random number sums. Using this method, the objective function can be converted to a QUBO format, which allows for quantum annealing computation.

## Methods

### Combinatorial random number sums^46^

**Figure 2:**
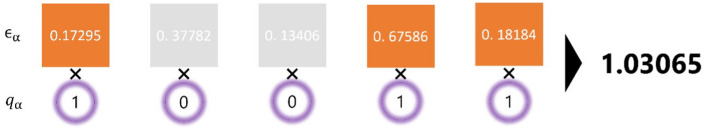
Image of “combinatorial random number sums”.

To perform an annealing analysis, the elastic strain energy and potential energy of a truss structure must be expressed in the format of QUBO. First, since displacements and cross-sectional areas, which are design variables of the analysis of actual trusses, are real numbers, they should be represented in the binary variables. For this purpose, real numbers are expressed as the combinatorial random number sums^46^. An image of this approach is shown in Fig. 2.

The real number *r* in the range of the constant $-a - a$ is represented by a combination of *N* qubits $q_{\alpha }$ that take zeros or ones, as in the following equation:

2 $$\begin{aligned} r=a\left\{ \left( 2 \sum _{\alpha \in N} \varepsilon _{\alpha } q_{\alpha } / \sum _{\alpha \in N} \varepsilon _{\alpha }\right) -1\right\} , \end{aligned}$$

where $\varepsilon _{\alpha }$ is a uniformly distributed random number between 0 and 1 generated by a classical computer. In this study, 16 binary variables, i.e., qubits are used for each of the *x*, *y*, and *z* components of one displacement or one cross-sectional area.

### Conversion of elastic strain energy to QUBO

The total strain energy of a truss structure is equal to the sum of the elastic strain energies of the trusses of the structure. The sum of the elastic strain energies can be formulated in the format of QUBO to allow the quantum annealing analysis of the truss structure.

**Figure 3:**
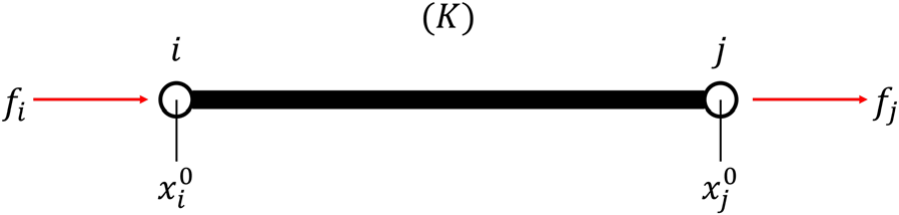
Truss element (*K*).

By considering the element (*K*) bounded by nodes *i* and *j* as shown in Fig. 3, we obtain the elastic strain energy $E^{(K)}$ as

3 $$\begin{aligned} E^{(K)}=k^{(K)}\left( \left\| {\varvec{x}}_{i}-{\varvec{x}}_{j}\right\| -l_{i j}^{(K)}\right) ^{2}, \end{aligned}$$

where $k^{(K)}$ is the $i-j$ spring constant of the element (*K*), ${\varvec{x}}_{i}$ is the coordinate vector of node *i*, and $l_{i j}^{(K)}$ is the natural length of the element (*K*) with nodes *i* and *j*.

Next, an infinitesimal deformation approximation is applied to the elastic strain energy. If we let ${\varvec{x}}_{i}^{0}$ be the natural position of *i* and ${{\varvec{u}}_{i}}$ be the displacement of *i*, we have ${\varvec{x}}_{i}={\varvec{x}}_{i}^{0}+{{\varvec{u}}_{i}}$. An infinitesimal deformation approximation of Eq. (3) gives the following equation:

4 $$\begin{aligned} \begin{aligned} E^{(K)}&=k^{(K)}\left( \left\| x_{i}-x_{j}\right\| -l_{i j}^{(K)}\right) ^{2} \\&= k^{(K)}\left( \sqrt{ \left( x_{i}^{0 x}-x_{j}^{0 x}+u_{i}^{x}-u_{j}^{x}\right) ^{2} +\left( x_{i}^{0 y}-x_{j}^{0 y}+u_{i}^{y}-u_{j}^{y}\right) ^{2} +\left( x_{i}^{0 z}-x_{j}^{0 z}+u_{i}^{z}-u_{j}^{z}\right) ^{2}} -l_{i j}^{(K)}\right) ^{2} \\&\simeq k^{(K)} \left( \sqrt{ {l_{i j}^{(K)}}^{2} +2\left( x_{i}^{0 x}-x_{j}^{0 x}\right) \left( u_{i}^{x}-u_{j}^{x}\right) +2\left( x_{i}^{0 y}-x_{j}^{0 y}\right) \left( u_{i}^{y}-u_{j}^{y}\right) +2\left( x_{i}^{0 z}-x_{j}^{0 z}\right) \left( u_{i}^{z}-u_{j}^{z}\right) } -l_{i j}^{(K)}\right) ^{2} \\&\simeq k^{(K)}\left( \left( x_{i}^{0 x}-x_{j}^{0 x}\right) \left( u_{i}^{x}-u_{j}^{x}\right) /l_{i j}^{(K)}\right. \left. +\left( x_{i}^{0 y}-x_{j}^{0 y}\right) \left( u_{i}^{y}-u_{j}^{y}\right) /l_{i j}^{(K)}\right. \left. +\left( x_{i}^{0 z}-x_{j}^{0 z}\right) \left( u_{i}^{z}-u_{j}^{z}\right) /l_{i j}^{(K)}\right) ^{2}, \end{aligned} \end{aligned}$$

where $(x_{i}^{0 x}-x_{j}^{0 x})$, $(x_{i}^{0 y}-x_{j}^{0 y})$, $(x_{i}^{0 z}-x_{j}^{0 z})$, $l_{ij}^{(K)}$, and $k^{(K)}$ are constants, while $(u_{i}^{x}-u_{j}^{x})$, $(u_{i}^{y}-u_{j}^{y})$, $(u_{i}^{z}-u_{j}^{z})$ are variables. Note that in the deformation of the equation from the second to the third line, terms above the second order with respect to displacement were neglected because of the small deformation. The third to fourth lines of the equation transformation utilized the fact that for sufficiently small $x=2((x_{i}^{0 x}-x_{j}^{0 x})(u_{i}^{x}-u_{j}^{x})+(x_{i}^{0 y}-x_{j}^{0 y})(u_{i}^{y}-u_{j}^{y})+(x_{i}^{0 z}-x_{j}^{0 z})(u_{i}^{z}-u_{j}^{z}))/l_{ij}$, the following equation holds.

5 $$\begin{aligned} \sqrt{1+x} \simeq 1 + \frac{x}{2} \end{aligned}$$

By using combinatorial random number sums in Eq. (2) for $\left( u_{i}^{x}-u_{j}^{x}\right)$, $\left( u_{i}^{y}-u_{j}^{y}\right)$ and $\left( u_{i}^{z}-u_{j}^{z}\right)$ , we can convert this approximate expression (4) to a QUBO expression.

### Conversion of potential energy to QUBO

Here, we let *W* be the load applied to the *i*th node. The potential energy difference $\Delta U$ after the *y*-directional deformation becomes

6 $$\begin{aligned} \Delta U=Wu_{i}^{y}. \end{aligned}$$

By using combinatorial random number sums in Eq. (2) for $u_{i}^{y}$, we can convert this expression to a QUBO expression.

### Optimization analysis of truss structure by quantum annealing

The optimization process is divided into two processes: the first is a deformation analysis and the second is a cross-sectional area optimization. These processes are iteratively repeated to obtain a truss structure that maximizes stiffness and minimizes displacement.

The spring constant $k^{(K)}$ of the truss element (*K*) is expressed in terms of the cross-sectional area as

7 $$\begin{aligned} k^{(K)}=C^{(K)}A^{(K)}/l_{i j}^{(K)}, \end{aligned}$$

where $C^{(K)}$ is Young’s modulus and $A^{(K)}$ is the cross-sectional area of the truss element (*K*)

By rewriting Eq. (4) using Eq. (7), we obtain the following equation:

8 $$\begin{aligned} \begin{aligned} E^{(K)}&=C^{(K)}A^{(K)}/l_{i j}^{(K)} \left\{ \left( x_{i}^{0 x}-x_{j}^{0 x}\right) \left( u_{i}^{x}-u_{j}^{x}\right) /l_{i j}^{(K)}+\left( x_{i}^{0 y}-x_{j}^{0 y}\right) \left( u_{i}^{y}-u_{j}^{y}\right) /l_{i j}^{(K)}\right. \\&\quad \left. +\left( x_{i}^{0 z}-x_{j}^{0 z}\right) \left( u_{i}^{z}-u_{j}^{z}\right) /l_{i j}^{(K)}\right\} ^{2}. \end{aligned} \end{aligned}$$

To make the cross-section $\Delta A^{(K)}$ a variable, it is expressed as a binary variable as in the following equation:

9 $$\begin{aligned} \begin{aligned} \Delta A^{(K)}=N\left\{ \left( 2(\sum _{\alpha \in N}2^{\alpha -1}q_{\alpha })/\sum _{\alpha \in N}2^{\alpha -1}\right) -1\right\} , \end{aligned} \end{aligned}$$

where $\Delta A^{(K)}$ takes the value $-N \leqq \Delta A^{(K)} \leqq N$, and $\Delta A^{(K)}$ is expressed as a combination of powers of 2, thereby allowing values of $\Delta A^{(K)}$ to be changed gradually.

To find the truss structure that maximizes stiffness to minimize the deflection under the condition of constant weight, the Hamiltonian of Eq. (8) is inverted from a positive to negative value as

10 $$\begin{aligned} \begin{aligned} H&=-C^{(K)}(A^{(K)}+\Delta A^{(K)})/l_{i j^{(K)}} \left\{ \left( x_{i}^{0 x}-x_{j}^{0 x}\right) \left( u_{i}^{x}-u_{j}^{x}\right) /l_{i j}^{(K)}\right. \left. +\left( x_{i}^{0 y}-x_{j}^{0 y}\right) \left( u_{i}^{y}-u_{j}^{y}\right) /l_{i j}^{(K)}\right. \\&\quad \left. +\left( x_{i}^{0 z}-x_{j}^{0 z}\right) \left( u_{i}^{z}-u_{j}^{z}\right) /l_{i j}^{(K)}\right\} ^{2}. \end{aligned} \end{aligned}$$

Since the Hamiltonian for the cross-sectional area calculation (Eq. (10)) and the Hamiltonian for the balance calculation (Eq. (8)) are inversely positive and negative respectively, they cannot be optimized simultaneously in a single calculation. Therefore, in this study, the displacement and cross-sectional area calculations are performed separately, and both calculations are repeated for structural optimization.

**Figure 4:**
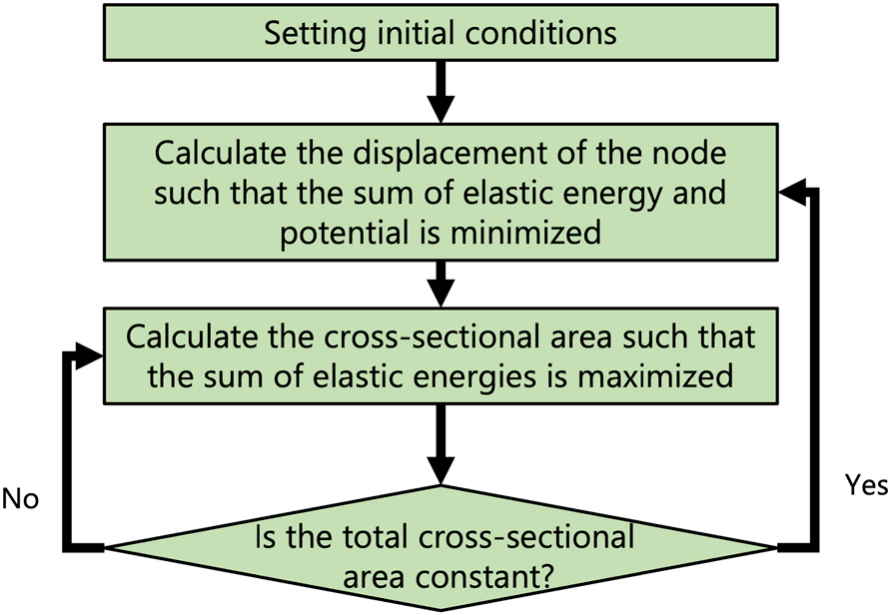
Flowchart of optimization analysis.

Step 1 First, initial conditions are set. The initial conditions for this study are shown in Tables 1 and 2.
Step 2 The balance of the truss structure is calculated. In this case, the displacements are taken as variables, and the cross-sectional areas as constants. Since the summation of the elastic strain energy and potential energy of the truss should be minimized, the Hamiltonian *H* used in this calculation can be obtained from Eqs. (8) and (6) as
  11 $$\begin{aligned} H=H_{1}+H_{2}, \end{aligned}$$
  where $H_{1}$ and $H_{2}$ are expressed as
  12 $$\begin{aligned} \begin{aligned} H_{1}&=C^{(K)}A^{(K)}/l_{i j}^{(K)} \left( \left( x_{i}^{0 x}-x_{j}^{0 x}\right) \left( u_{i}^{x}-u_{j}^{x}\right) /l_{i j}^{(K)}+\left( x_{i}^{0 y}-x_{j}^{0 y}\right) \left( u_{i}^{y}-u_{j}^{y}\right) /l_{i j}^{(K)}\right. \\&\left. \quad +\left( x_{i}^{0 z}-x_{j}^{0 z}\right) \left( u_{i}^{z}-u_{j}^{z}\right) /l_{i j}^{(K)}\right) ^{2} \end{aligned} \end{aligned}$$
  and
  13 $$\begin{aligned} H_{2}=Wu_{i}^{y}, \end{aligned}$$
  respectively. Here, if the displacements of node *i* i.e., $u_{i}^{x}$, $u_{i}^{y}$ and $u_{i}^{z}$ are not fixed, $A^{(K)}$ is set as the value of the initial cross-sectional area. Here, $u_{i}^{x}$, $u_{i}^{y}$ and $u_{i}^{z}$ are represented by a combination of binary variables using combinatorial random number sums in Eq. (2). When the displacements of node *i* i.e., $u_{i}^{x}$, $u_{i}^{y}$, and $u_{i}^{z}$ are fixed, they are set as $u_{i}^{x}=0$, $u_{i}^{y}=0$ and $u_{i}^{z}=0$. The displacements are updated to the value obtained.
Step 3 The value of the cross-sectional area is searched to maximize the elastic strain energy of the truss structure under the condition that the summation of the cross-sectional areas of the trusses is constant. In this case, the cross-sectional areas are taken as variables and displacements as constants. The following penalty function is added to the Hamiltonian to set the sum of the cross-sectional areas of the truss structure, denoted as $A_{sum}$:
  14 $$\begin{aligned} H_{N1}=\left( \sum A^{(K)}-A_{sum}\right) ^{2}. \end{aligned}$$
  If the value of the summation of the cross-sectional areas of all elements changes from the constant of $A_{sum}$, $H_{N1}$ increases and the solution is removed from the candidates.
  Using Eq. (10), we express the Hamiltonian *H* used in this calculation as
  15 $$\begin{aligned} H=H_{3}+H_{N1}, \end{aligned}$$
  where
  16 $$\begin{aligned} \begin{aligned} H_{3}&=-C^{(K)}(A^{(K)}+\Delta A^{(K)})/l_{i j}^{(K)}\left( \left( x_{i}^{0 x}-x_{j}^{0 x}\right) \left( u_{i}^{x}-u_{j}^{x}\right) /l_{i j}^{(K)}+\left( x_{i}^{0 y}-x_{j}^{0 y}\right) \left( u_{i}^{y}-u_{j}^{y}\right) /l_{i j}^{(K)}\right. \\&\quad \left. +\left( x_{i}^{0 z}-x_{j}^{0 z}\right) \left( u_{i}^{z}-u_{j}^{z}\right) /l_{i j}^{(K)}\right) ^{2} \end{aligned} \end{aligned}$$
  Here, $\Delta A^{(K)}$ is represented by a combination of binary variables using combinatorial random number sums in Eq. (2). The cross-sectional areas are updated to the value obtained.

Repeat steps 2 and 3. (Fig. 4) The Leap Hybrid Solver from D-Wave is used for these analyses^50^.

## Results

### Deformation analysis of truss structures by quantum annealing

**Figure 5:**
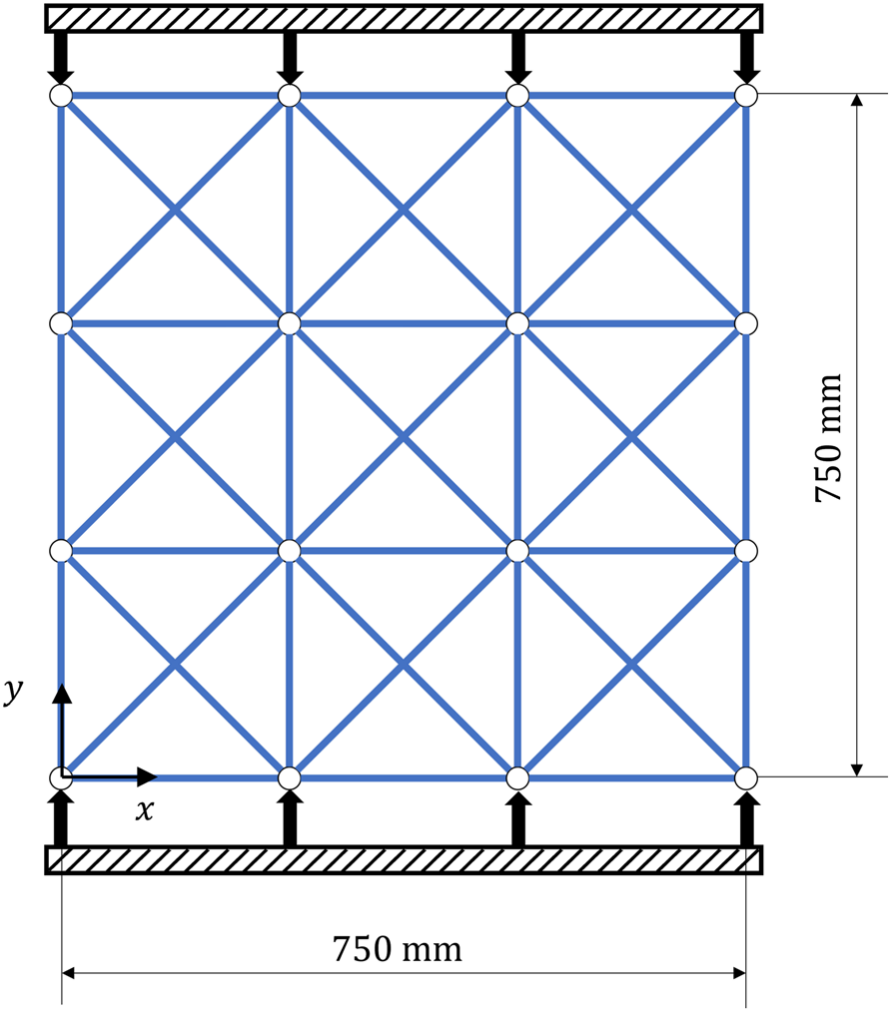
Truss model for deformation analysis.

Deformation analysis was performed by applying the forced displacement and compression to a truss consisting of 42 truss elements as shown in Fig. 5. The analysis conditions are shown in Table 1.

**Table 1:** Analysis conditions for deformation.

Number of nodes	16
Length of one truss element	$250\,\textrm{mm}$
Maximum value of change in displacement	$25\,\textrm{mm}$
Cross-sectional area	$50\,\mathrm {mm^2}$
Young’s modulus	$5\,\textrm{MPa}$
Forced displacement	$12.5\,\textrm{mm}$
Number of binary variables used for one displacement	16

### Calculation condition for structural optimization

#### Two-dimensional problem

**Figure 6:**
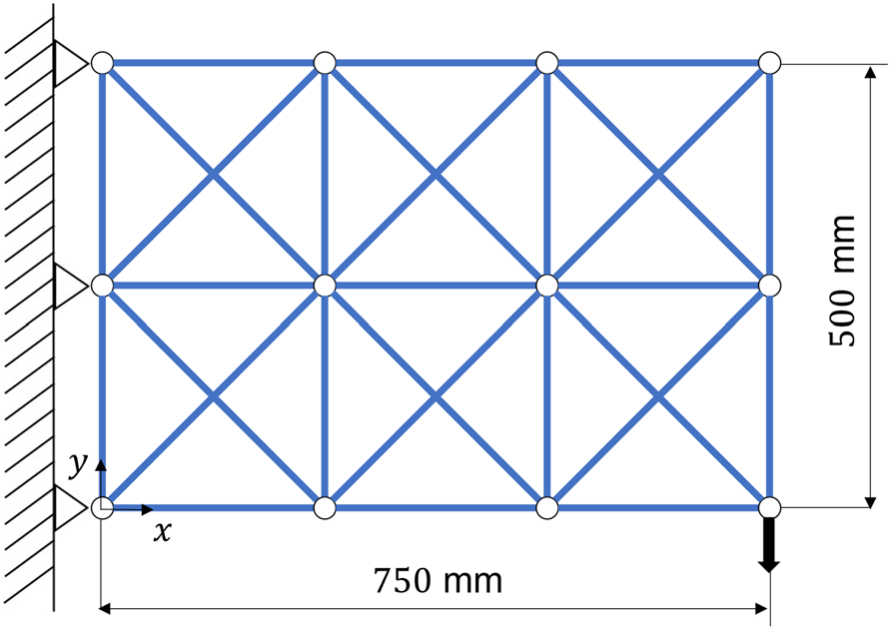
Two-dimensional truss model for structural optimization.

Optimization calculations are performed for a wall-hung truss structure consisting of 29 truss elements. The node at the left end is fixed and a load is applied to the lower right, as shown in Fig. 6. The analysis conditions are shown in Table 2.

Now, we find the truss structure that maximizes stiffness to minimize deflection under the condition of constant weight.

**Table 2:** Analysis conditions for two-dimensional truss optimization.

Number of nodes	12
Length of one truss element	$250\,\textrm{mm}$
Maximum value of change of displacement	$25\,\textrm{mm}$
Initial cross-sectional area	$50\,\mathrm {mm^2}$
Young’s modulus	$5\,\textrm{MPa}$
Load	$10\,\mathrm N$
Number of binary variables used for one displacement	16
Number of binary variables used for one cross-sectional area	5

#### Three-dimensional problem

**Figure 7:**
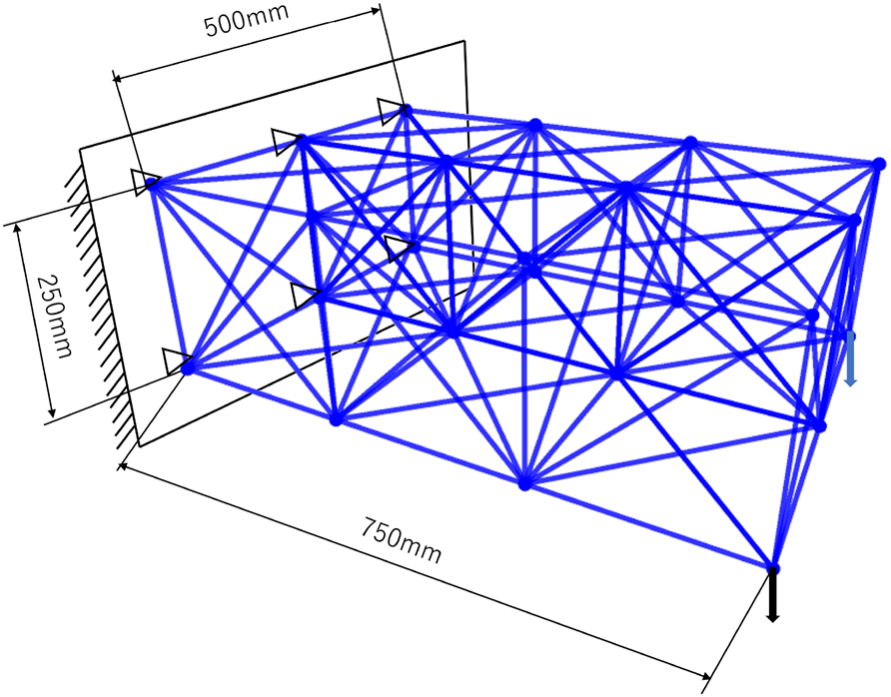
Three-dimensional truss model for structural optimization.

Structural optimization was performed for a wall-hung truss consisting of 60 elements as shown in Fig. 7. The analysis conditions are shown in Table 3.

**Table 3:** Analysis conditions for three-dimensional truss optimization.

Number of nodes	24
Length of one truss element	$250\,\textrm{mm}$
Initial cross-sectional area	$50\,\mathrm {mm^2}$
Maximum value of change of cross-sectional area	$15\,\mathrm {mm^2}$
Young’s modulus	$5\,\textrm{MPa}$
Load	$10\,\mathrm N$
Number of binary variables used for one displacement	16
Number of binary variables used for one cross-sectional area	5

### Result of deformation analysis of truss structure by quantum annealing

**Figure 8:**
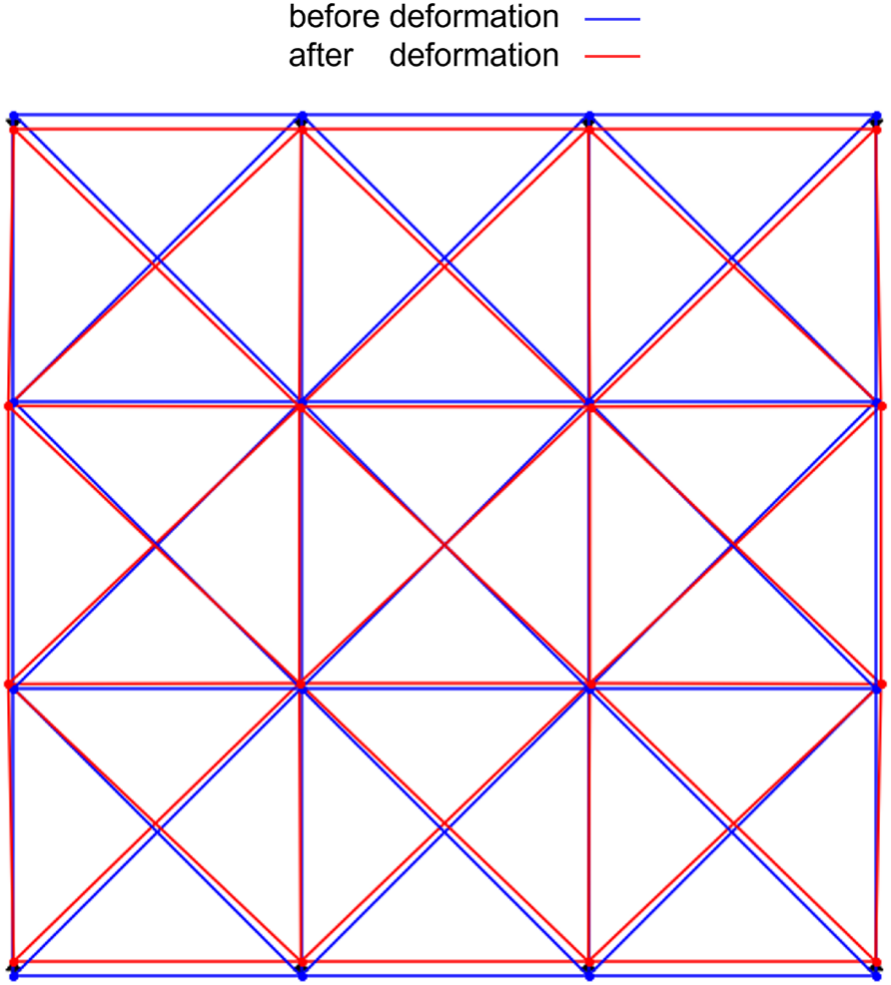
Two-dimensional compression analysis result.

The result of the analysis of compression is shown in Fig. 8. The blue line represents the before deformation, and the red line represents the after deformation. It was found that the compression in the *y*-direction is accompanied by an elongation in the *x*-direction. This result confirms the usefulness of quantum annealing using the formulated Hamiltonian for the analysis of truss deformation.

### Result of optimization analysis of truss structure by quantum annealing

#### Two-dimensional problem

**Figure 9:**
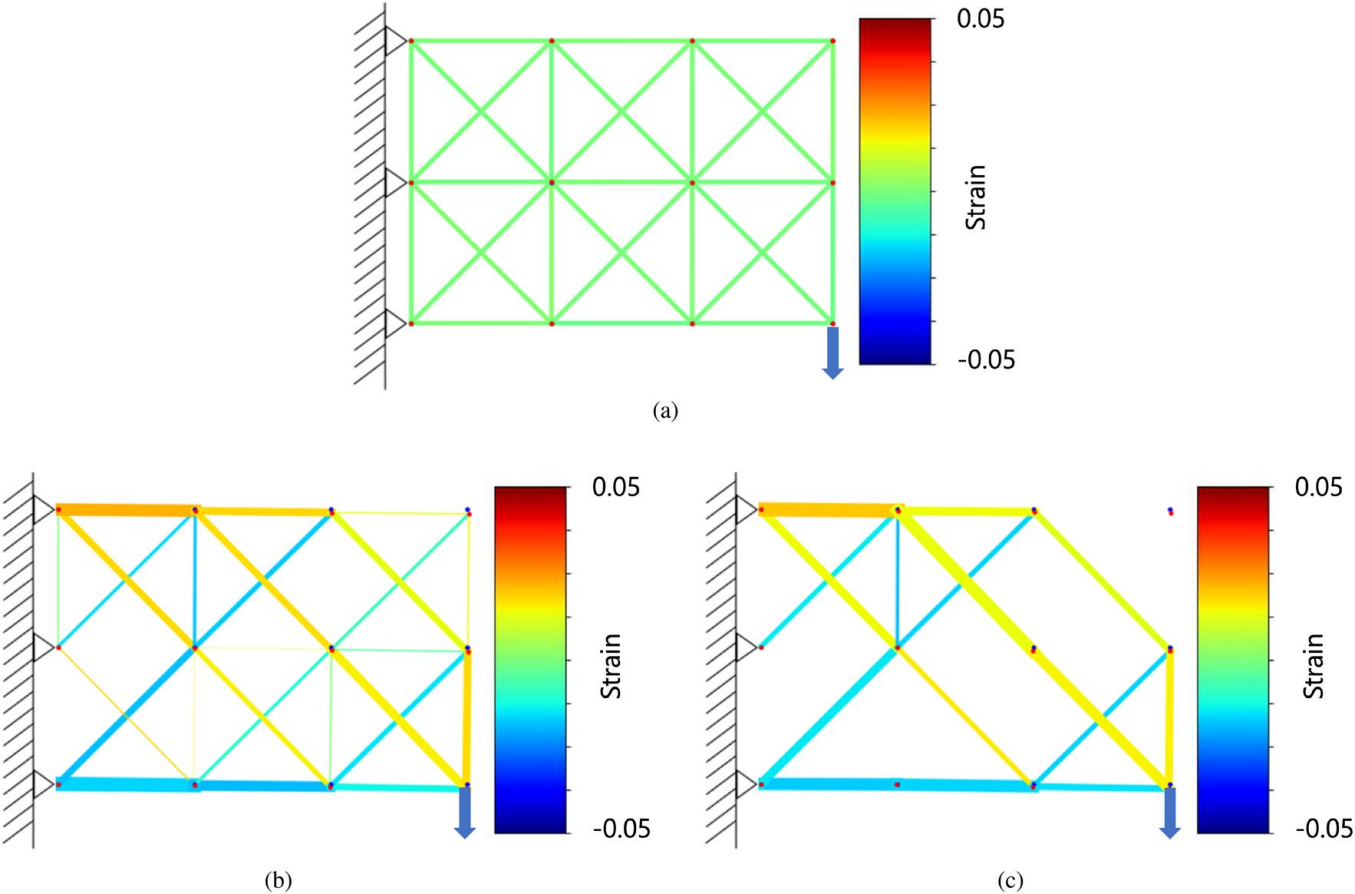
Two-dimensional truss optimization results. (**a**) Truss structure under initial condition. (**b**) Truss structure at step 15. (**c**) Truss structure at step 30.

The two-dimensional optimization analysis was performed in 30 steps. The initial condition is shown in Fig. 9a, the results at step 15 are shown in Fig. 9b, and the results at step 30 as shown in Fig. 9c. The strain of each truss element is shown in color contours. The blue dots are the nodes before deformation and the red dots are the nodes after deformation. Similar to the results of the analysis of beam bending, the truss elements on the upper side of the structure are in tension and the truss elements on the lower side of the structure are in compression. The structure shows a tapered shape that is similar to the conventional optimal shape when loads are applied to the end side, as seen in conventional optimization methods. By setting a limit on the value of the cross-sectional area that can increase or decrease significantly at one time, we can repeatedly conduct the calculations while maintaining the relationship between the displacement and the cross-sectional area.

#### Three-dimensional problem

**Figure 10:**
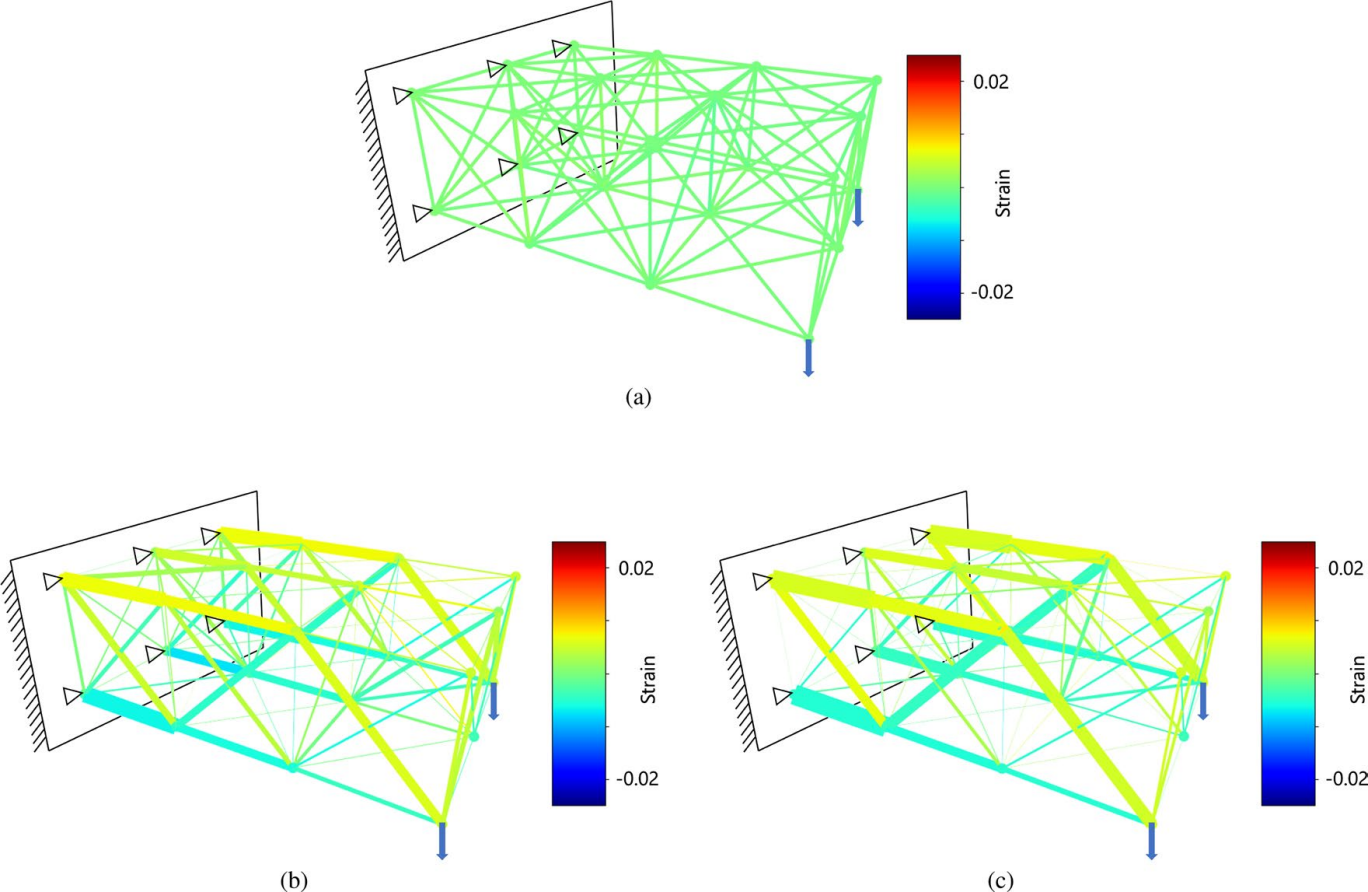
Three-dimensional truss optimization results. (**a**) Truss structure under initial condition. (**b**) Truss structure at step 15. (**c**) Truss structure at step 30.

The three-dimensional analysis was performed in 30 steps. The initial condition is shown in Fig. 10a, the result at step 15 is shown in Fig. 10b, and the result at step 30 is shown in Fig. 10c. The strain in each truss element is shown in color contours. The three-dimensional truss also shows a structure that tapers on the loaded-end side.

## Discussion

**Figure 11:**
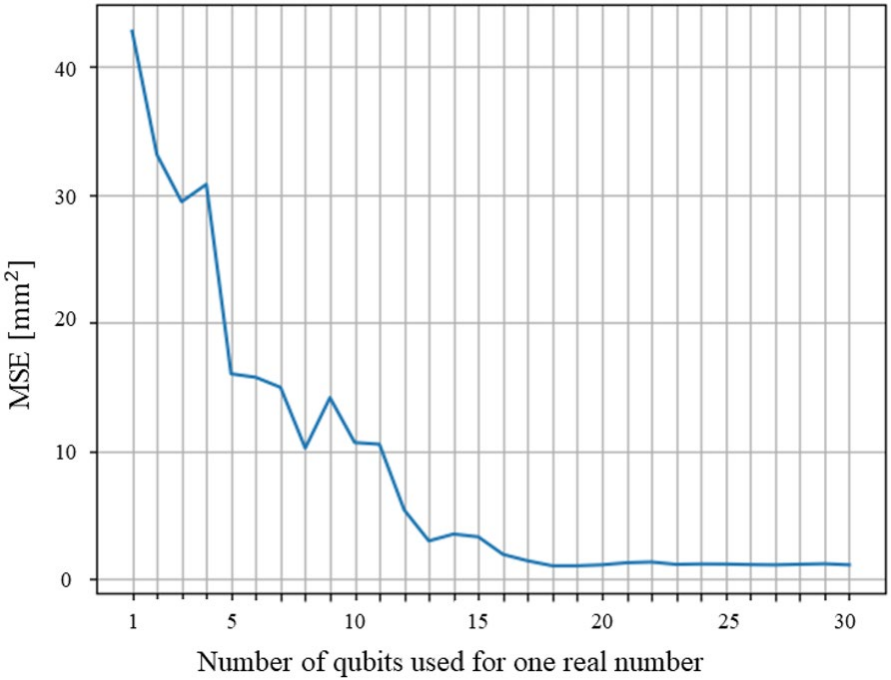
Relationship between the mean square error of the exact solution and the result of the analysis by the proposed method and the number of qubits used for one real number.

In this study, we proposed a method to perform deformation analysis and topology optimization analysis for truss structures by quantum annealing. We proposed a Hamiltonian with elastic strain energy for deformation analysis. For optimization analysis, we proposed a Hamiltonian and a method to solve the optimization of the structure. By expressing nodal displacements as binary variables and using the elastic strain energy and position energy of the truss as objective functions, we confirmed that the proposed method enables us to analyze the truss deformation with quantum annealing. Figure 11 shows a graph plotting how the mean square error from the exact solution *MSE* changes as the number of qubits used for the single real number is changed.

The mean square error was calculated as follows:

17 $$\begin{aligned} MSE = \frac{1}{2N_\mathrm{{nodes}}} \sum _i^{N_\mathrm{{nodes}}} \Vert {\varvec{u}}_i^*-{\varvec{u}}_i\Vert ^2 \end{aligned}$$

where $N_\mathrm{{nodes}}$ is the number of nodes of the truss structure, ${\varvec{u}}_i^*$ is the displacement of node *i* calculated by the proposed method, and ${\varvec{u}}_i$ is the displacement of node *i* calculated by finite element analysis. In the deformation analysis, increasing the number of qubits tended to approach the exact solution. The mean square error between the analytical results and the exact solution using the proposed method was 0.641 $\%$ compared to the maximum deformation $12.5^2$ $\textrm{mm}^2$. This confirms the validity of the Hamiltonian formulation of elastic energy in Eq. (4)

**Figure 12:**
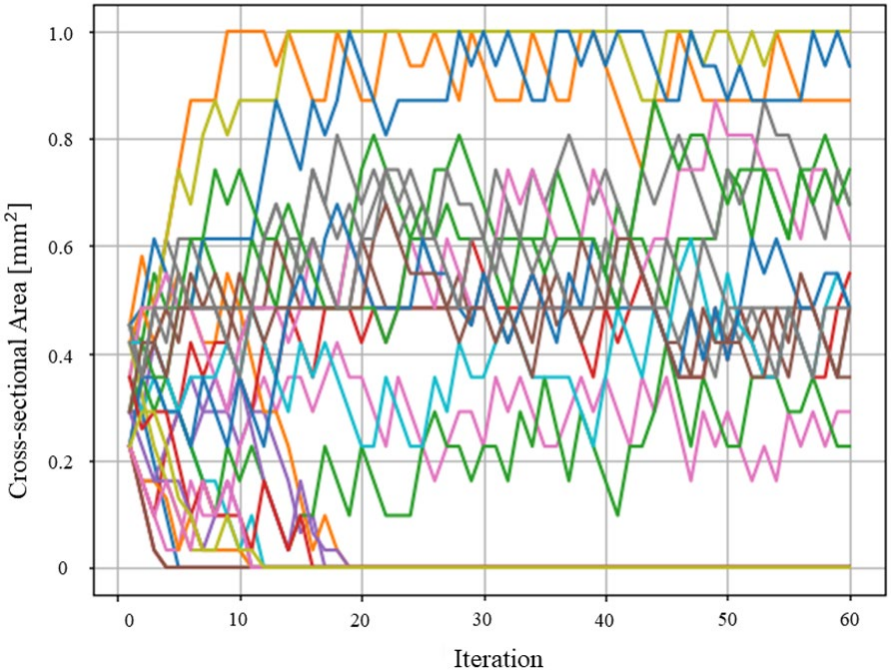
History of cross-sectional area of two-dimensional truss.

**Figure 13:**
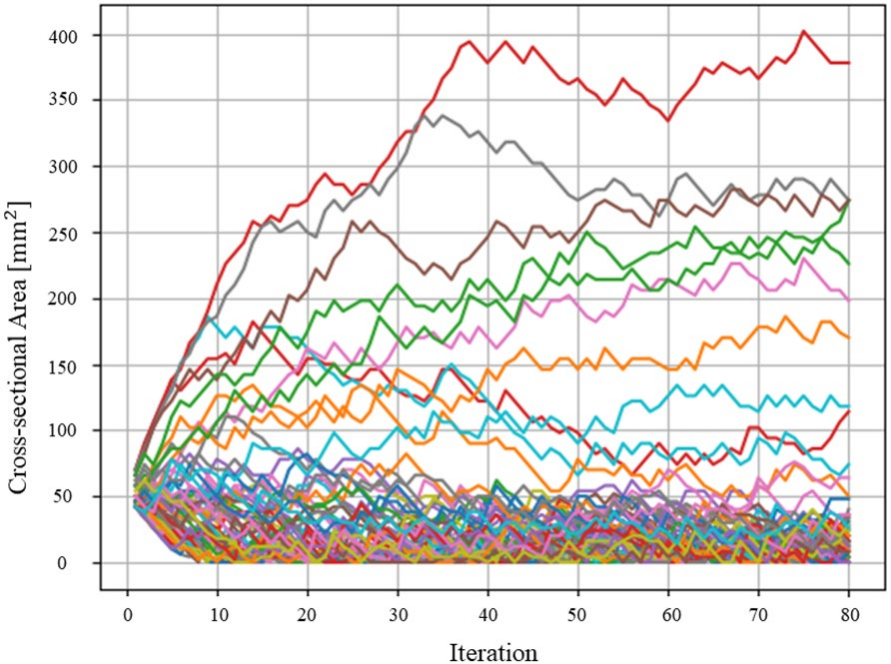
History of cross-sectional area of three-dimensional truss.

**Figure 14:**
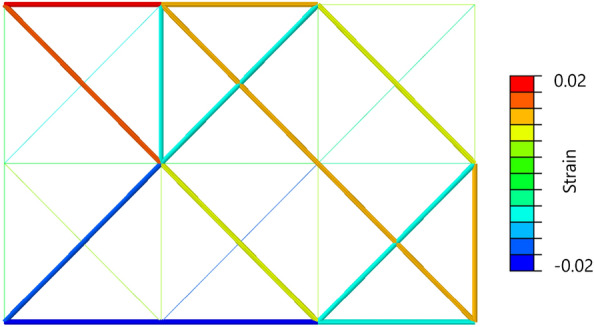
Structure by conventional method.

**Figure 15:**
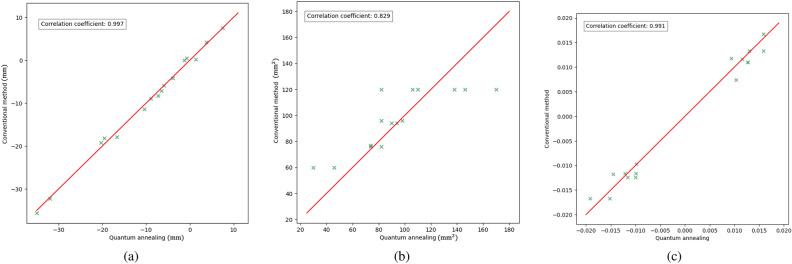
Quantum annealing vs. conventional method. (**a**) Nodal displacement. (**b**) Cross-sectional area. (**c**) Strain.

It also became possible to optimize truss structures with quantum annealing by expressing the increasing and decreasing values of the cross-sectional area as a binary variable. Figure 12 depicts a graph plotting the history of the cross-sectional areas of each element of the two-dimensional truss. As the evolution of the cross-sectional areas reached a near-equilibrium state by the 30th step, the structure at that point was considered the optimal solution. Figure 13 also depicts a graph plotting the history of the cross-sectional areas of each element of the three-dimensional truss. As the evolution of the cross-sectional areas reached a near-equilibrium state by the 30th step, the structure at that point was considered the optimal solution. Optimization calculations were performed on a classical computer under the same conditions as the optimization calculations performed in this study and their comparison was made: concerning Reference^51^, optimization of a two-dimensional truss structure using the optimality criteria method was implemented using Abaqus and Isight. Figure 14 depicts a result of the conventional method. The external shapes of both results show a similar trend. The nodal displacement, cross-sectional area, and strain values for each result were compared. Figure 15a–c to show the correlations. The correlation coefficients calculated for both results were 0.998 for nodal displacement, 0.829 for cross-sectional area, and 0.991 for strain. Note that trusses that are almost completely missing or nodes with no connected trusses are excluded from the comparison. All of the correlations are high, indicating that the structure obtained by the present method is valid.

In the current D-Wave full quantum annealing machine, the Advantage, approximately 5000 qubits are available. If 16 qubits are used per real variable, the machine can treat problems with around 300 degrees of freedom (DOFs). The current D-wave Leap Hybrid Solver can handle up to 1,000,000 bits, which allows problems with approximately 60,000 DOFs under the condition of 16 bits for real variable representation. Fixstars Amplify Annealing Engine, a digital annealer, manages up to 260,000 bits, enabling us to treat problems with up to 16,000 DOFs. Regarding the computation time, each optimization step took approximately 3 seconds using the current D-wave Leap Hybrid Solver in this study. It is expected that the performance of quantum devices will be improved and be able to handle larger-scale problems in the future.

In future work, we are considering extending the method proposed in this study to structural optimization of 2D and 3D continuum elements. In addition, as an extension of truss structure analysis by quantum annealing, we are considering developing a method for structural analysis of trusses with nonlinearities by quantum annealing.

The advantage of this research is that quantum annealing is expected to be able to search for the global optimal solution faster than conventional methods when the problem size is large.

An important aspect of solving large-scale problems is how to develop the method to effectively combine the classical computation and quantum computation. In nonlinear problems with continuum elements, for example, it is expected that a low-resolution solution obtained by quantum annealing will be used as an initial condition for a high-resolution solution in classical computing. This approach has the potential to accelerate the convergence in complex nonlinear large problems. Moreover, sparse matrices are frequently utilized in the structural analysis. The incorporation of variables that generate the elastic energy into the coupled qubits in quantum annealing machines is anticipated to enhance computational efficiency. Cooperation between hardware and applications is expected.

### Supplementary Information

Supplementary Information.

## Data Availability

The datasets used and/or analysed during the current study available from the corresponding author on reasonable request.

## Code availability

Code for analysis is provided as part of the replication package. It is available in Supplementary Material.
