# Supplementary material for: Development of optimization method for truss structure by quantum annealing
Source: Sci Rep. 2024 Jun 16;14:13872. doi: 10.1038/s41598-024-64588-2 (PMC11180109; doi:10.1038/s41598-024-64588-2)
Supplement: Supplementary file 1 — Supplementary Information. [file 41598_2024_64588_MOESM1_ESM.pdf]

Supplementary Material

The following is the python code used in this study.

```
%pip install amplify==0.12.1
# Does not work with version 1.0 or later.

import os
from datetime import datetime
import numpy as np
import matplotlib as mpl
from matplotlib import pyplot as plt
from amplify import BinaryPoly, gen_symbols, BinaryQuadraticModel, Solver,
↪  decode_solution
from amplify.client import FixstarsClient

token = "your token"

#turss data
cNsqrt = [4, 3]
point = [[[0, 0], [0, 0]], [[0, 1], [0, 0]], [[0, 2], [0, 0]]]
length = [0.25, 0.25]

dirname=f"{datetime.now():%Y%m%d%H%M%S}"
os.mkdir(dirname)

Young = 5   # MPa
Load = 10   # N
PreArea = 50 # mm^2 Initial cross-sectional area

ran = 0.07 # Range of combinatorial random number sums
qPer = 16 # Number of divisions in the combinatorial random number sums

# Setting of nodal id.
(cNx,cNy) = cNsqrt
cN = cNx * cNy
crsp = [ (i,j) for i in range(cNx) for j in range(cNy) ]
toid = lambda i,j: i * cNy + j

lconst = []
a = 0
for x1,y1 in crsp:
    for x2,y2 in crsp:
        if x1==x2 and y1 == y2:
            continue
        if np.linalg.norm([x2-x1,y2-y1])<1.5:
            if toid(x1,y1) > toid(x2,y2):
                continue
            xx1=length[0]*x1
            xx2=length[0]*x2
            yy1=length[1]*y1
            yy2=length[1]*y2
            lconst.append( [(x1,y1),(x2,y2),(xx1,yy1),(xx2,yy2),
            ↪  np.linalg.norm([xx2-xx1,yy2-yy1]),a ] )
            plt.plot((xx1,xx2),(yy1,yy2))
            a=a+1
plt.axis('square')
```

```python
plt.show()

Area = [PreArea] * len(lconst)

def solve(model,timeout=1000):
    client = FixstarsClient()
    client.token = token
    client.parameters.timeout = timeout
    solver = Solver(client)
    result = solver.solve(model)
    print(client.parameters.timeout)
    for solution in result:
        print(f"energy = {solution.energy}\nvalues = {solution.values}")
    return result

def deformation(Area,timeout=1000):

    qN = qPer * cN * 2
    qcCoef = np.random.rand(cN, 2, qPer)

    # combinatorial random number sums
    q = gen_symbols(BinaryPoly, qN)
    Deltas = []
    for c in range(cN):
        dli = []
        for ax in range(2):
            dax = 0
            for i in range(qPer):
                dax += qcCoef[c, ax, i] * q[c * 2 * qPer + ax * qPer + i]
            dax = (2 * dax / sum(qcCoef[c][ax]) - 1) * ran
            dli.append(dax)
        Deltas.append(dli)

    # boundary condition
    for (x, y), (dx, dy) in point:
        c1id = toid(x, y)
        Deltas[c1id][0] = dx
        Deltas[c1id][1] = dy

    Ham = 0

    # boundary energy
    for (x1, y1), (x2, y2), (xx1, yy1), (xx2, yy2), lg, a in lconst:
        if Area[a] == 0:
            Area[a] = 0.02
        c1id = toid(x1, y1)
        c2id = toid(x2, y2)
        dL = 0
        dL += (xx2 - xx1) * (Deltas[c2id][0] - Deltas[c1id][0]) / lg
        dL += (yy2 - yy1) * (Deltas[c2id][1] - Deltas[c1id][1]) / lg
        Ham += Young * Area[a] / 2 / lg * (dL) ** 2

    # gravity
    c1id = toid(3, 0)
    Ham += Load * Deltas[c1id][1]
```

```
    model = BinaryQuadraticModel(Ham)

    print("Use qbit:", qN, model.num_logical_vars)
    print("Use Num of Grid Point", cN)

    result = solve(model,timeout)

    # Real-valued restoration of results
    vals = decode_solution(q, result.solutions[0].values)
    rdeltas = np.sum(vals[0 : qPer * cN * 2].reshape(cN, 2, qPer) * qcCoef, axis=-1)
    rdeltas = (rdeltas / np.sum(qcCoef, axis=-1) * 2 - 1) * ran

    # External Force
    for (x, y), (dx, dy) in point:
        c1id = toid(x, y)
        rdeltas[c1id][0] = dx
        rdeltas[c1id][1] = dy


    print(rdeltas)
    print(rdeltas.shape)
    return rdeltas

def cul_strain(rdeltas):
    strain = []
    for (x1, y1), (x2, y2), (xx1, yy1), (xx2, yy2), lg, a in lconst:
        c1id = toid(x1, y1)
        c2id = toid(x2, y2)
        dx1,dy1 = rdeltas[c1id,0],rdeltas[c1id,1]
        dx2,dy2 = rdeltas[c2id,0],rdeltas[c2id,1]
        strain.append((np.linalg.norm([xx2+dx2-xx1-dx1,yy2+dy2-yy1-dy1]) \
        -np.linalg.norm([xx2-xx1,yy2-yy1]))/np.linalg.norm([xx2-xx1,yy2-yy1]))
    return strain

def print_deformation(loop,rdeltas,Area,strain):
    plt.figure(figsize=(8,8))

    norm = mpl.colors.Normalize(-0.05, 0.05)
    cmap = plt.get_cmap("jet")

    for (x1,y1),(x2,y2),(xx1,yy1),(xx2,yy2),lg,a in lconst:
        c1id = toid(x1,y1)
        c2id = toid(x2,y2)
        dx1,dy1 = rdeltas[c1id,0],rdeltas[c1id,1]
        dx2,dy2 = rdeltas[c2id,0],rdeltas[c2id,1]
        plt.plot((xx1+dx1,xx2+dx2),(yy1+dy1,yy2+dy2),color = cmap(norm(strain[a])),
         ↪  linewidth = 0.05*Area[a])

    for x1,y1 in crsp:
        c1id = toid(x1,y1)
        dx1,dy1 = rdeltas[c1id,0],rdeltas[c1id,1]
        plt.plot([x1*length[0]],[y1*length[1]],"b.",markersize=8)
        plt.plot([x1*length[0]+dx1],[y1*length[1]+dy1],"r.",markersize=8)

    plt.axis('square')
    plt.tight_layout()
```

```
    plt.savefig(os.path.join(dirname, "%d_a.png" %loop))


def optimize(rDeltas,timeout=1000):

    # List of powers of two
    d = 3
    bin = []
    for j in range(d):
        da = 2**j
        bin.append(da)

    qN = d * len(lconst)

    q = gen_symbols(BinaryPoly, qN)

    qArea = []
    for ax in range(len(lconst)):
        dax = 0
        for i in range(d):
            dax = dax + bin[i] * q[ax * d + i]
        if Area[ax] > 8:
            dax = (4 * dax) - 8 + Area[ax]
        else:
            dax = (4 * dax)
        if Area[ax] <= 0.02:
            dax = 0
        qArea.append(dax)

    Ham = 0
    ene2 = 0

    for (x1, y1), (x2, y2), (xx1, yy1), (xx2, yy2), lg, a in lconst:
        c1id = toid(x1, y1)
        c2id = toid(x2, y2)
        dL = 0
        dL += (xx2 - xx1) * (rDeltas[c2id][0] - rDeltas[c1id][0]) / lg
        dL += (yy2 - yy1) * (rDeltas[c2id][1] - rDeltas[c1id][1]) / lg
        ene2 += qArea[a] / lg * (dL) ** 2
    Ham -= 100 * ene2

    Ham += 10000.0 * (sum(qArea) - 50 * len(lconst)) ** 2

    model = BinaryQuadraticModel(Ham)

    print("Use qbit:", qN, model.num_logical_vars)
    print("Use Num of Grid Point", cN)

    result = solve(model,timeout)

    # Real-valued restoration of results
    vals = decode_solution(q, result.solutions[0].values)
    qArea = np.sum(vals[0:qN].reshape(len(lconst), d) * bin, axis=-1)
    qArea = qArea * 4 + Area - 8
    for ze in range(len(lconst)):
        if Area[ze] < 8:
```

```
            qArea[ze] = qArea[ze] + 8 - Area[ze]
        if Area[ze] <= 0.02:
            qArea[ze] = 0
    print("sum area =", sum(qArea))
    print(qArea)

    ene2 = 0
    return qArea

def print_optimize(loop,rdeltas,Area):
    plt.figure(figsize=(8,8))
    for (x1,y1),(x2,y2),(xx1,yy1),(xx2,yy2),lg,a in lconst:
        c1id = toid(x1,y1)
        c2id = toid(x2,y2)
        dx1,dy1 = rdeltas[c1id,0],rdeltas[c1id,1]
        dx2,dy2 = rdeltas[c2id,0],rdeltas[c2id,1]
        plt.plot((xx1+dx1, xx2+dx2), (yy1+dy1, yy2+dy2), "r", linewidth = 0.05 *
        ↪  Area[a])

    for x1, y1 in crsp:
        c1id = toid(x1,y1)
        dx1, dy1 = rdeltas[c1id, 0], rdeltas[c1id, 1]
        plt.plot([x1 * length[0]], [y1 * length[1]],"b.",  markersize=8)
        plt.plot([x1 * length[0] + dx1], [y1 * length[1]+dy1],"r.", markersize=8)

    plt.axis('square')
    plt.tight_layout()
    plt.savefig(os.path.join(dirname, "%d_b.png" %loop))

for loop in range(30):
    rdeltas = deformation(Area,3000)
    strain = cul_strain(rdeltas)
    print_deformation(loop,rdeltas,Area,strain)

    Area = optimize(rdeltas,3000)
    print_optimize(loop,rdeltas,Area)
```
